# The use of bidirectional barbed suture in the treatment of a complete common calcanean tendon rupture in a dog: Long‐term clinical and ultrasonographic evaluation

**DOI:** 10.1002/ccr3.2287

**Published:** 2019-07-10

**Authors:** Kevin Frame, Oded Ben‐Amotz, Renee Simpler, Josh Zuckerman, Ron Ben‐Amotz

**Affiliations:** ^1^ Veterinary Specialty and Emergency Center Bluepearl Veterinary Partners Philadelphia Pennsylvania; ^2^ Rambam 80, Hand and Microsurgery Unit Healthcare Campus Haifa Israel; ^3^ Cape Cod Veterinary Specialists Buzzards Bay Massachusetts

**Keywords:** canid, veterinary, orthopaedics

## Abstract

The canine common calcanean tendon can be repaired successfully using a modified Kessler knotless barbed technique. A long‐term ultrasound follow‐up showed improved and increased normal tendon fibrillar echotexture and homogeneity.

## 1 INTRODUCTION

Use of bidirectional‐barbed suture for primary repair of a traumatic common calcanean tendon rupture in a 1 year‐old Pitbull allowed for an excellent outcome with progressive improvement in tendon morphology evident on ultrasound imaging at 12 and 48 months following surgery.

The common calcanean tendon (Achilles tendon) is comprised of the paired gastrocnemius tendons, the conjoined tendons of the gracilis, semitendinosus, and biceps femoris muscles, and the superficial digital flexor tendon. Partial or complete tears are typically seen following a traumatic incident and are classified based upon duration of the injury (acute vs chronic).^1^, ^2^ Affected animals are typically presented with a visible skin laceration and a plantigrade stance.^2^

The current treatment recommendation for an acute rupture of the common calcanean tendon is primary tenorrhaphy. During the first 4 to 5 days following repair, the tendon ends will lose holding power.^3^ This period is followed by a progressive increase in strength over the next 2 weeks, although it has been reported that the presence of suture material may prolong the inflammatory phase.^4^ The suture material selected must meet the need for strength during this period while also minimizing the potential for physical damage and tissue reactivity. The use of monofilament, synthetic, slowly absorbable, or nonabsorbable materials is recommended.^3^

The linear arrangement of collagen fibers in a normal tendon provides little holding strength for simple suture patterns. Specialized suture patterns are required to resist pullout when the tendon is placed under tension. The locking loop, 3‐loop pulley, Krackow, and double Bunnell‐Mayer suture patterns have all been used successfully for primary tenorrhaphy.^5^, ^6^, ^7^, ^8^ Additional procedures to augment the primary repair using the semitendinosus muscle and tensor fascia lata have also been reported.^9^, ^10^, ^11^ The presence of a knot in the suture increases cross‐sectional area at the repair site and serves as a weak point in the suture. These factors have also been shown to be associated with an increase in gliding resistance.^12^ The use of barbed suture material eliminates the need for a knot and the barb‐tendon interaction throughout the repair provides a more even load distribution.^13^

Ultrasound has been shown to be an excellent diagnostic method for imaging lesions of the common calcanean tendon and differentiation of total ruptures, partial ruptures, and those comprising muscular tears.^14^ On ultrasonographic examination, a normal common calcaneal tendon appears as a moderately echogenic, homogenous structure with parallel hyperechoic lines (fibrillar echotexture).^15^ Ultrasound also has been used to document the healing process and, in humans, has been suggested as a guide for physiotherapy in patients undergoing surgery for common calcanean tendon rupture.^16^ Eight weeks after surgery, replacement tissue begins to grow in the normal longitudinal direction and a decrease in diameter of the tendon is accompanied by the reappearance of the characteristic tendinous fibrillar echotexture. However, after the healing process is completed, the tendon remains less homogeneous in comparison with the unaffected tendon for long periods and nonabsorbable suture material remains visible as hyperechoic foci.^15^

The use of barbed suture in tendon repair may have several advantages over traditional suture materials including increased tensile strength, less repair‐site bunching, decreased cross‐sectional area, the absence of knots as weak points, decreased gliding resistance, and decreased adhesion formation.^15^ We propose the use of barbed suture for the repair of a common calcaneal tendon rupture will provide a favorable outcome in this case as documented by postoperative clinical and ultrasonographic findings.

## 2 CASE HISTORY

A one‐year‐old, 17 kg, spayed female Pitbull mix was presented with a chief complaint of left hind limb lameness following direct trauma involving sharp metal framing. The patient had no history of lameness in the affected limb prior to this event. At the time of admission, the dog was able to ambulate with a plantigrade stance. A deep skin laceration in the region of the common calcaneal tendon was observed, and a complete transverse tendon laceration was suspected. Orthogonal radiographs of the tarsal region revealed discontinuity of the opaque soft tissue margin of the calcaneal tendon with interspersed fat opacity. No bony abnormalities were identified. An acute, complete, transverse common calcaneal tendon laceration was diagnosed based on the clinical presentation, physical examination, and radiographic findings. A preoperative complete blood count and serum biochemical profile were within normal limits.

## 3 SURGICAL PROCEDURE

**Figure 1:**
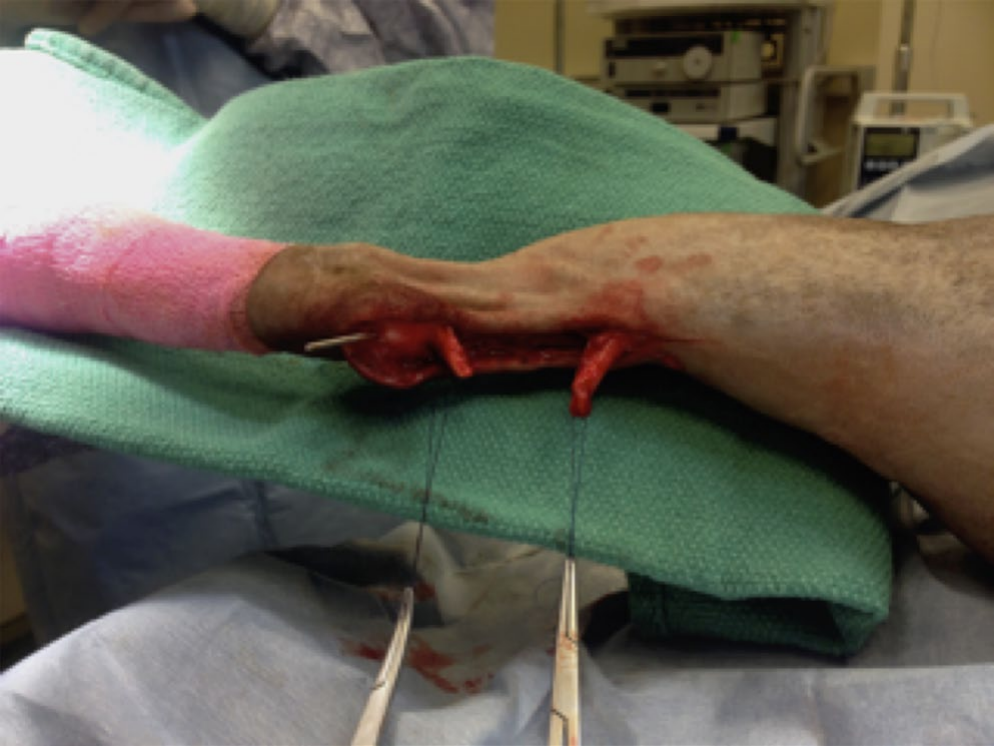
Intraoperative exposure of the lacerated tendon ends

Exploration of the injured left common calcaneal tendon was performed via a lateral para‐tendon approach. The laceration was extended proximally and distally over the torn tendon to allow adequate exposure of the tendon ends (Figure 1). With the tarsocrural joint held in extension, an 0.062 k‐wire was placed temporarily from the proximal aspect of the calcaneal tuber to the distal aspect of the tibial diaphysis in a proximocranial direction. A 2.5 mm drill hole was made through the calcaneal tuber in a proximocranial direction, ending in the distal aspect of the tibial diaphysis just distal to the k‐wire. The hole was tapped using a 3.5 mm tap, and a 3.5 mm cortical screw was placed from the plantar aspect of the calcaneus ensuring that the tarsocrural joint was avoided. The k‐wire was removed.

**Figure 2:**
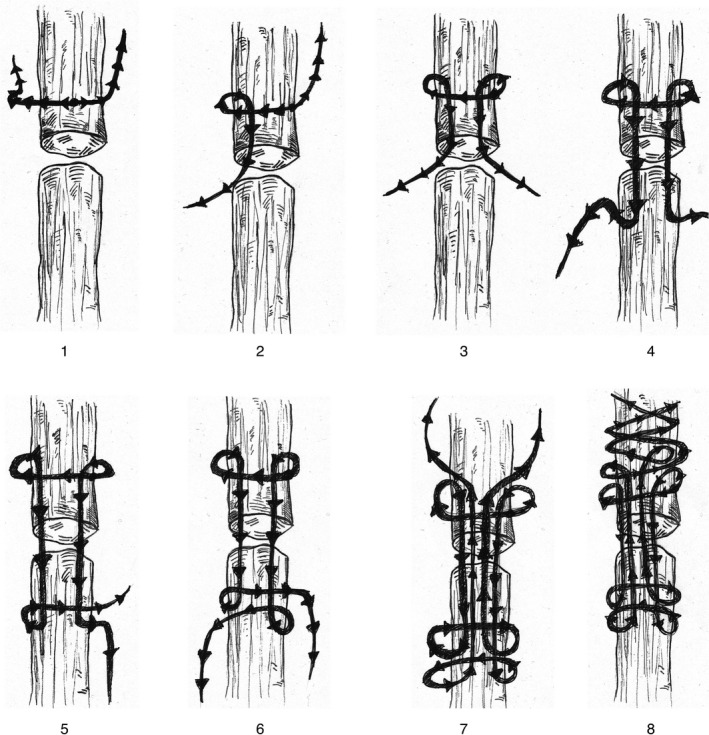
Barbed suture method of common calcanean tendon repair using a modified Kessler pattern. (A) Bidirectional barb design of Quill SRS features sutures barbed placed in opposing direction on either side of the tendon segment. One segment of barbed suture is advanced transversely through the stump of the tendon until opposing barbs engage and prevent further advancement. (2) The first leg of the initial core suture is advanced longitudinally, exiting at the cut tendon end. (3) The second leg of the initial core suture is advanced longitudinally from the opposite side of the tendon stump. (4) Both sutures are then advanced longitudinally through and out of the distal stump. (5) The first leg is passed transversely through the stump, proximal to its exit site. (6) The second leg is also passed similarly in the opposite direction. (7) Both legs are passed longitudinally across the apposed tendon ends to exit from the opposite tendon stump. (8) Each end of the barbed suture makes an additional 3 subsequent transverse passes

Repair of the damaged tendon was achieved using 2‐0 polypropylene bidirectional barbed suture (Quill^™^ 2‐0) in a modified Kessler suture pattern (Figure 2). The subcutaneous tissue was closed using 3‐0 Monoderm^™^ bidirectional barbed suture (Quill^™^ 3‐0).

## 4 OUTCOME

Following surgery, the limb was placed in a full bi‐valve cast (Vetcast^™ ^Plus). Exercise was limited to controlled leash walks for the first 8 weeks at which point the bone screw was removed and the limb was placed in a lateral splint for an additional 4 weeks. This was followed by 4 weeks in a custom‐made hinged brace (Thera‐Paw^™^). Serial palpation revealed subjective focal improvement in tendon thickness throughout the healing period, with only mild to moderate thickening noted at the time of brace removal (16 weeks postoperatively). Four months postoperatively the dog had returned to full activity without appreciable lameness. Seven months postoperatively, palpation of the tendon revealed very mild focal thickening.

## 5 FOLLOW‐UP IMAGING

**Figure 3:**
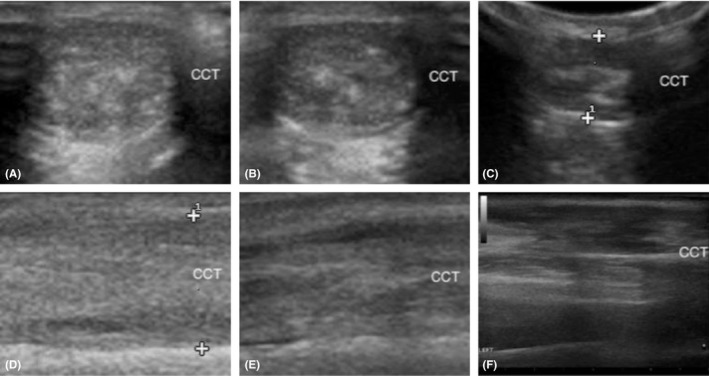
Transverse (A, B, C) and longitudinal (D, E, F) ultrasound images of the calcaneal tendon made at 7 mo (A, D), 12 mo (B, E), and 48 mo (C, F) postoperatively. Note the hyperechoic structure within the tendon in image C consistent with the nonabsorbable barbed suture. No suture material was observed outside of the healing tendon, eliminating a potential source interference with tendon gliding. Slight improvement in fibrillar echotexture of the tendon is evident in the longitudinal images made at 12 and 48 mo (E, F). CTT, common calcanean tendon

**Figure 4:**
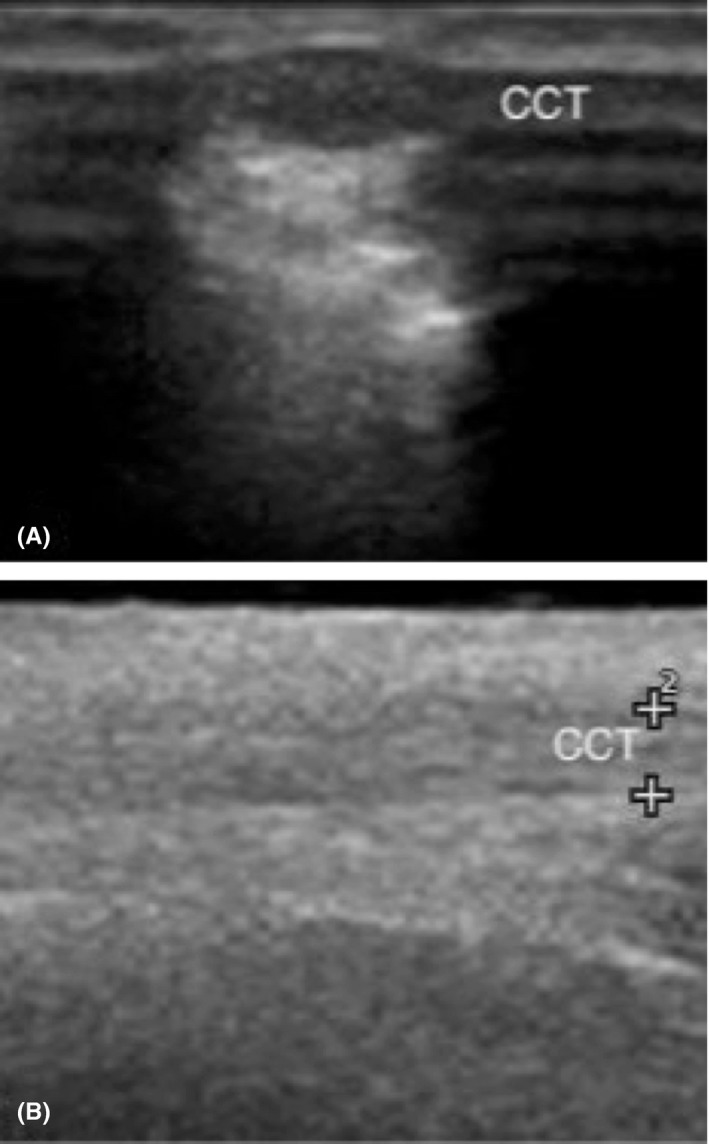
Transverse (A) and longitudinal (B) ultrasound images of the uninjured contralateral calcaneal tendon. Note the moderately echogenic, homogenous structure with parallel hyperechoic lines (fibrillar echotexture). CTT, common calcanean tendon

Ultrasound of the left common calcaneal tendon was performed at 7 months, 12 months, and 48 months postoperatively using a Siemens P300 Acuson (Siemens Healthcare Gmbh) and a 6‐18 MHz LA435 linear transducer (Figure 3). The left common calcaneal tendon was thicker compared to that of the contralateral limb (Figure 4), measuring 0.9 cm thick at 7 months, 0.8 cm thick at 12 months, and 0.7 cm thick at 48 months in the transverse plane (contralateral tendon: 0.2‐0.3 cm thick). In the longitudinal plane, the thickness of the affected tendon was 1.1 cm, 0.8 cm, and 0.8 cm, respectively. In all 3 examinations, the left common calcaneal tendon appeared inhomogeneous with loss of the normal fibrillar echotexture. However, progressive improvement was noted at 12 and 48 months, with increased normal fibrillar echotexture and improved homogeneity.

## 6 DISCUSSION

The successful use of knotless barbed suture has been reported in the veterinary literature for use in laparoscopic gastropexy, enterotomy, gastrotomy, and intradermal wound closure.^17^, ^18^, ^19^, ^20^, ^21^, ^22^, ^23^, ^24^, ^25^ Quill^™^ suture was introduced initially as a barbed monofilament suture made from polypropylene, nylon, or glycolic acid derivatives. The suture has a curved, diamond‐pointed needle on each end of the strand. The length of suture is divided into two halves, each of which contains barbs oriented in a spiral fashion away from the needle on the same side. The mid‐point of the suture length can be determined by identifying a segment that has no barbs, or by feeling for an increase in resistance as the suture is gently pulled through the tissue. This along with other commercially available barbed suture has been successfully tested in many biomechanical human and animal tendon models.^12^, ^27^, ^28^, ^29^, ^30^, ^31^, ^32^, ^33^, ^34^, ^35^, ^36^, ^37^, ^38^, ^39^, ^40^, ^41^, ^42^

In the human literature, the use of at least four specialized barbed sutures for flexor tendon repair was described in several studies between 1945 and 1968 but were not widely adopted.^27^ Work by McKenzie^28^ in 1967 involving barbed suture for the repair of flexor tendons in cadaveric models established early data regarding tensile strength of barbed suture, finding it comparable to more traditional techniques. This was confirmed in a recent meta‐analysis of barbed suture in flexor tendon repair compared to conventional methods.^43^ Multiple studies have also demonstrated superior tensile strength, gap formation, and final failure force of knotless barbed suture techniques compared to conventional methods,^27^, ^29^, ^30^, ^31^ though this is also dependent on suture pattern and size.^32^, ^33^ One study found that use of a knotless barbed suture technique was biomechanically inferior to a 4‐strand modified Kessler technique in a chicken model.^34^ Regardless of repair technique, suture knots have been consistently found to be the point of tensile weakness in tendon repair.^27^, ^35^, ^36^ When located within the tendon repair site, knots may impede tendon healing, stimulate an inflammatory response, impede revascularization, induce a foreign body response, and hinder gliding by increasing tendon cross‐sectional area. Similarly, knots that are located external to the repair may also impair gliding within the tendon sheath and are a potential nidus for adhesion formation.^12^, ^27^, ^34^, ^44^

Barbed polypropylene sutures differ from their smooth counterparts in that the barbs are cut into the actual strand, thus decreasing overall suture diameter. It has been demonstrated that the breaking strength of 2‐0 barbed polypropylene suture is closest to, and not significantly different from, that of smooth 3‐0 polypropylene suture. The barbed suture was also significantly stiffer and had less elongation than any of the smooth suture sizes tested in that study.^45^ A recent in vitro veterinary study compared the ultimate tensile strength and load to 1 and 3 mm gap formation between smooth and knotless barbed polypropylene sutures for canine gastrocnemius tendon repair.^37^ Thirty‐three paired bone‐tendon units were repaired with either 3‐0 smooth or 0 knotless barbed polypropylene suture using a three‐loop pulley pattern and tested under single‐cycle tensile loading until failure occurred. The study found that smooth polypropylene suture exhibited a significantly higher ultimate tensile strength compared to the barbed suture. The barbed repairs also required significantly lower loads to induce 1 and 3 mm gaps.^37^

A study of human Achilles tendon repairs also reported a comparatively lower peak load to failure when barbed suture was used.^38^ Similar results were demonstrated in an ex vivo study evaluating performance and resistance to gap formation of nonabsorbable, barbed, monofilament suture compared to nonabsorbable, smooth, monofilament polypropylene suture.^39^ Three‐loop pulley and modified Bunnell‐Mayer patterns were used in transected cadaveric superficial digital flexor tendons, and the smooth polypropylene suture was consistently superior in load performance when compared to unidirectional barbed suture. This study also demonstrated that the three‐loop pulley pattern was more effective in resisting gap formation compared to the Bunnell‐Mayer pattern.^39^

In contrast to the recent studies,^37^, ^39^ our case report suggests that barbed suture can be used as an effective alternative to traditional suture for repair of a common calcaneal tendon rupture. In the current case report, a modified Kessler suture technique was used rather than a three‐loop pulley suture technique. The modified Kessler technique was chosen as it allows for an increased number of tendon‐suture anchor points. It functions by creating a loop that grasps longitudinal bundles of tendon fibers (Figure 2). This loop further tightens and pinches the fibers as load is increased. It has been reported that the primary mode of failure affecting the traditional modified Kessler suture technique is due to suture pullout rather than suture breakage or knot failure.^34^ It is possible that by increasing the size of each loop and by using a locking suture pattern, both the quantity of tendon fibers grasped as well as the interface between tendon fibers and suture material may be increased, ultimately resulting in an overall stronger repair and decreased risk of pullout. Further biomechanical studies would be necessary to validate this theory.

The size of the suture used in the present study differs from what has been previously reported. We used a 2‐metric polypropylene suture in contrast to the 4‐metric suture used in the in vitro study.^37^ Although the suture size in our study is smaller and therefore inherently weaker, this was an in vivo report and the patient's repair was protected by a calcaneotibial screw and variable external coaptation throughout the healing period that may explain the differences between the studies. A biomechanical study that performed cyclic testing under physiologic loads would have been more representative of the in vitro conditions under which our patient was subjected.

Numerous studies have demonstrated that a greater number of anchor points and suture strands significantly increase overall repair strength.^12^, ^27^, ^31^, ^33^, ^46^ Zeplin et al^31^, ^33^ showed that for two‐strand repairs the traditional modified Kirchmayr‐Kessler technique was stronger than the knotless technique; however, no difference in maximal load to failure was found between four‐strand techniques. McClellan et al^40^ showed that a four‐strand knotless technique had equivalent strength and reduced cross‐sectional area at the repair site compared to a traditional four‐strand technique and that this repair was significantly stronger than a traditional two‐strand Kessler technique. A four‐strand knotless barbed suture technique has also been shown to have higher resistance to gap formation and higher final force to failure compared to a conventional four‐strand cross‐locked cruciate (Adelaide) technique.^30^ Thus, it is likely that the use of more suture strands in knotless barbed suture techniques may further improve their strength and performance.

Postoperative immobilization of the tarsus is potentially more important than the specific suture pattern chosen for successful common calcanean tendon healing.^3^ Many different techniques have been described including the use of a calcaneotibial screw (CTS), transarticular external fixator (TESF), and/or external coaptation.^3^ In clinical use, it has been advised that a full cylinder cast or cranial half cast is used in combination with a CTS to decrease the risk of cyclic loading of the screw and premature screw breakage, especially in large dogs.^47^ Neilsen and Pluhar^48^ found no difference in outcome between TESF and the use of a splint or cast alone for common calcanean tendon injuries in dogs, though TESF resulted in longer surgical time and higher initial cost. In a small case series, Worth et al^49^ found that the use of a cast alone resulted in a poorer outcome than using a CTS with casting for common calcanean tendon injuries in working dogs. The authors observed fewer cast‐related complications when the CTS was combined with a cast, likely because rigid immobilization of the tarsocrural joint prevents hock motion within the cast thereby decreasing rubbing and the risk of secondary skin complications.

Use of a CTS provides a low‐cost option for short‐term immobilization, avoiding the complications associated with a TESF. Complications associated with a CTS include screw bending and/or breakage, potentially necessitating screw replacement. In one retrospective study of 38 dogs undergoing screw and cast immobilization following common calcanean tendon repair, 3 (8%) experienced complications associated with the screw.^10^ Almost all postoperative complications in the CTS group were associated with the external coaptation, and overall complication rates for CTS were significantly lower than for those stabilized with external fixation or a cast alone.

Traditionally, it has been recommended that a calcaneotibial screw be kept in place for 6‐8 weeks, at which time it can be removed and a splinted support bandage placed for an additional 2‐4 weeks.^3^ At 6 weeks after tenorrhaphy, a tendon has achieved 56% of its original strength and is therefore able to withstand the normal forces associated with limited exercise.^3^ Despite these findings, the optimal postoperative method and duration of tarsal immobilization in dogs is currently unknown. Research into ligamentous injuries in humans has suggested that prolonged rest or immobilization could in fact have adverse effects on tissue repair and healing and that early mobilization may actually stimulate repair and decrease the time required for adequate rehabilitation.^50^, ^51^, ^52^

An experimental study in dogs that compared postoperative management following knee ligament repair found that immobilization for longer than 6 weeks resulted in considerable damage to the articular cartilage.^53^ The ideal fixation method may be a hinged TESF which allows for controlled progressive increases in tendon loading and a gradual increase in tarsal flexion.^54^ However, a hinged TESF adds complexity and cost to the initial stabilization and is associated with a high complication rate.

Tendon and ligament healing directly benefits from early mobilization of the joint. New tendon collagen should ideally be exposed to certain amounts of strain or load 3 weeks following repair to encourage the fibers to develop in the appropriate orientation. Controlled load or strain starting at this time has been shown to result in more rapid return of tendon strength compared to longer periods of immobilization.^55^ This benefit however must be balanced against the risk of early disruption or elongation. Therefore, protection of the repair for a period greater than 3 weeks is still generally recommended.^3^

The immobilization method selected in this case was chosen based on the anticipated healing times following tendon repair, the concern regarding the dog and owner's compliance, as well as the reported lower complication rate when a calcaneotibial screw is used in conjunction with external coaptation compared to external coaptation alone. A hinged brace was also used later in the recovery period to allow a gradual increase in tendon loading and tarsal range of motion similar to that allowed by a hinged TESF. 56 The period of postoperative immobilization in this case was likely excessive, but was chosen based on historical recommendations and the use of a suture material not previously described in a clinical case. It is possible that earlier mobilization of the tarsus in this case may have resulted in improved or more rapid tendon healing evident on ultrasound examination. It is hoped that demonstrating the successful use and potential advantages of barbed suture in this case will encourage further investigation of its use followed by shorter postoperative immobilization periods, therefore minimizing the risks associated with prolonged joint immobilization.

A recent study evaluating the use of postoperative ultrasonography of the Achilles tendon in humans failed to identify a statistical correlation between long‐term ultrasonographic morphology and clinical outcome.^57^ However, a study in horses found that computerized ultrasonography was an effective tool for objective monitoring of healing superficial digital flexor tendons and provided reliable prognostication of repair quality.^58^ The ultrasonographic appearance of the common calcanean tendon in this report was observed to gradually trend toward that of the unaffected contralateral tendon over the 48‐month follow‐up period. At 48 months, the cross‐sectional diameter of the repaired tendon in the transverse plane remained much greater than that of the normal tendon (0.7 cm vs 0.2‐0.3 cm, respectively). Although they remained abnormal, inhomogeneity and fibrillar echotexture of the healed tendon were improved at 48 months. This is consistent with previous studies of Achilles tendon imaging following surgical repair in humans and canines in which the operated side exhibited a degree of thickening and inhomogeneity compared to the contralateral side for months to years postoperatively.^14^, ^16^, ^56^

One identified limitation of barbed suture material is that the barbs lock into tissue. Once the needle and suture are passed through the tendon, the suture and needle cannot be backed out. Recognizing this limitation, we recommend rehearsing the planned suture application and ensuring accurate needle positioning before completely pulling the needle through the tissue. We have demonstrated a successful repair of a canine common calcanean tendon with full return to function using a modified Kessler knotless barbed suture technique. By considering the different factors that influence the success of tendon repairs including suture size, suture pattern, and number of strands, the use of knotless barbed suture for canine common calcanean tendon repair could be a promising strategy and further clinical studies are indicated.

## AUTHOR CONTRIBUTIONS

Kevin Frame, BVSc: involved in manuscript preparation, revision, and research.Oded Ben‐Amotz, MD: involved in concept and design, surgeon. Renee Simpler, VMD, DACVR: involved in ultrasonographer, data acquisition, prepared the manuscript, and revised the manuscript. Josh Zuckerman, VMD, DACVS: prepared and revision the manuscript. Ron Ben‐Amotz, DVM, MS, DACVS, DECVS: involved in concept and design, surgeon, manuscript revision.
